# Neonatal adrenal hemorrhage presenting as late onset neonatal jaundice

**DOI:** 10.4103/0971-9261.59607

**Published:** 2009

**Authors:** Umar Amin Qureshi, Nisar Ahmad, Akhter Rasool, Suhail Choh

**Affiliations:** Department of Radiodiagnosis, Neonatology and Pediatrics, SKIMS, Srinagar, Kashmir, India

**Keywords:** Adrenal, jaundice, neonatal hemorrhage

## Abstract

Clinical manifestations of adrenal hemorrhage vary depending on the degree and rate of hemorrhage, as well as the amount of adrenal cortex compromised by hemorrhage. We report here a case of neonatal adrenal hemorrhage that presented with late onset neonatal jaundice. The cause of adrenal hemorrhage was birth asphyxia.

## INTRODUCTION

Neonatal adrenal hemorrhage (NAH) occurs during the first weeks of life. The unique vascular supply and large size compared to body weight makes adrenal vulnerable to hemorrhage during this period. The hemorrhage occurs most often after a traumatic delivery or a neonatal course complicated by hypoxia, hypotension, or coagulopathy. The commonest presentation is abdominal mass.[1] Neonatal jaundice is also common. However late onset jaundice is rarely reported as the primary presentation. We present a case of adrenal hemorrhage confirmed on contrast-enhanced computerized tomography (CECT) of abdomen and serial ultrasonography (USG) that presented with late onset neonatal jaundice. The cause of adrenal hemorrhage was birth asphyxia.

## CASE REPORT

**Figure 1:**
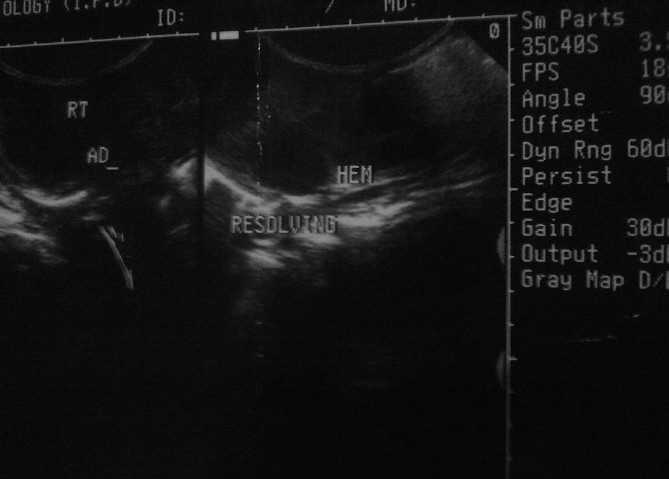
USG abdomen at 6 weeks revealing anechoic adrenal cyst

A 16-day-old, full term, exclusively breast fed, 3-kg male child presented with yellowish discoloration of body for 2 days with normal colored stools and urine. Systemic examination was normal except for icterus and a palpable mass in right lumbar region. His serum bilirubin was 14 mg/dl with direct fraction of 1 mg/dl. Laboratory studies revealed hemoglobin of 9 gm/dl and reticulocyte count of 5%. Red cells were normocytic. Glucose 6 phosphate dehydrogenase levels were normal. Septic profile, urine analysis, kidney, and liver function tests were normal. Both baby and mother had B+ blood group and infant was Coombs negative. USG abdomen revealed a solid echogenic mass between right lobe of liver and right kidney, which was displaced inferiorly, suggesting right adrenal mass. There was no paravertbral lymphadenopthy, no liver lesion was seen. CECT abdomen revealed a complex mass measuring 5×4 cm, with areas of dense attenuation and low attenuation within it, in the right adrenal region [Figure 1]. There was no enhancement in the post-contrast scan or calcification in the mass. The mass displaced the inferior vena cava anteriorly and right kidney inferiorly. The findings were compatible with adrenal hemorrhage. Coagulogram and 24-hour urinary vanillyl mandelic acid (VMA)/catecholamine levels were normal.

His records revealed that he was born of a nonconsanguineous marriage after an uneventful pregnancy per vaginum by breech. His Apgar score was 5/10 at 5 minutes and he required resuscitation in form of positive pressure ventilation with bag and mask for 5 minutes. He was admitted in intensive care with tachypnea. Arterial blood gas (ABG) revealed hypoxemia, C-reactive protein was negative, and hemogram was within normal limits. He was kept under oxygen hood on intravenous fluids (dextrose 10%), calcium gluconate, and antibiotics. At 12th hour of life, he developed neonatal seizures which were controlled with phenobarbitone. Repeat ABG at 24th hour of life were normal and respiratory distress settled. The blood culture was sterile. He was discharged on 4th day.

**Figure 2:**
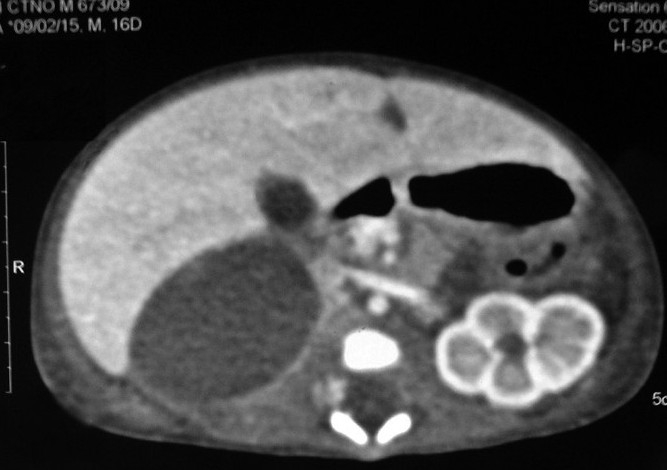
CECT abdomen showing large adrenal hemorrhage

His bilirubin levels continued to remain between 15 and 20 mg/dl till 6 weeks after which he became anicteric. Repeat USG abdomen at 4 weeks follow-up revealed 3×2 cm anechoic right adrenal cyst resulting from resolving right adrenal hemorrhage [Figure 2]. Hence, a diagnosis of birth asphyxia with hypoxic ischemic encephalopathy with adrenal hemorrhage with late onset neonatal jaundice was made.

## DISCUSSION

The incidence of adrenal hemorrhage ranges from 1.7 per 1000 autopsied newborn infants to approximately 3% of infants subjected to screening by abdominal USG.[2] The reason for such a high incidence being its large size compared to weight and a unique vascular supply. Relative to body weight, the gland is 10–20 times larger than in adults.[3] Fifty to sixty arterial branches from three suprarenal arteries supply each gland through a subcapsular plexus. This highly vascular plexus drains into medullary sinusoids via relatively few venous channels, thereby creating a potential “vascular dam.” Any condition leading to hypoxia may lead to shunting of blood flow to vital organs. Furthermore in times of physiologic stress, endogenous adrenocorticotropic hormone release further increases blood flow rates to critical organs by several folds resulting in hemorrhage into the gland. Hypoxia also causes damage to the endothelial cells, making them more prone to hemorrhage. Predisposing factors include birth asphyxia, birth trauma, septicemia, underlying tumor, and hypoprothrombinemia.[4]

Almost 70% NAH occurs on right side with 5–10% involvement in bilateral cases.[5] The usual explanations for susceptibility of the right adrenal gland is that it is more likely to be compressed between the liver and spine and, the right adrenal vein usually drains directly into the inferior vena cava, so it is prone to changes in venous pressure.

Clinical manifestations of adrenal hemorrhage can vary depending on the degree and rate of hemorrhage, as well as the amount of adrenal cortex compromised by hemorrhage. Although an isolated focal unilateral adrenal hemorrhage may present subclinically, massive bilateral adrenal hemorrhage may present in shock. Large unilateral or bilateral extensive bleeds may also present with palpable flank mass, anemia, jaundice, or scrotal hematoma. Addisonian crisis is rare in NAH because hemorrhage is predominantly subcapsular and adrenal insufficiency does not occur until at least 90% of adrenal tissue is destroyed.

Neonatal jaundice is common and occurs due to hemolysis from enclosed hemorrhage. Late onset neonatal jaundice is rarely the primary presentation. Thus possibility of adrenal hemorrhage should not be ignored as the cause of late onset indirect hyperbilirubinemia particularly in susceptible neonates.

USG is the modality of choice for the evaluation of an adrenal mass in a neonate. In neonates, the normal adrenal glands are clearly visualized at USG and consist of a hypoechoic cortex and a thin, echogenic medulla. The pattern of echogenicity of an adrenal hematoma depends on its age. An early-stage hematoma appears solid with diffuse or inhomogeneous echogenicity. As liquefaction occurs, the mass demonstrates mixed echogenicity with a central hypoechoic region and eventually becomes completely anechoic and cyst like. Calcification can appear as early as 1–2 weeks after hemorrhage. CECT scan is reserved for cases with doubt. Acute to subacute hematomas contain areas of high attenuation that usually range from 50 to 90 HU. An adrenal hematoma may calcify after 1 year. An organized chronic hematoma appears as a mass with a hypoattenuating center with or without calcifications. Such masses are termed adrenal pseudo cysts.

The closest differential diagnosis of adrenal hemorrhage is neuroblastoma particularly the cystic form. Determination of the urinary excretion of VMA is relevant, since an increase in VMA is virtually diagnostic of neuroblastoma. Over 90% of children with neuroblastoma will have elevated urinary excretion of catecholamine metabolites.[6] If the suprarenal mass is a purely sonolucent ovoid mass, if color doppler USG reveals no blood flow signal over the mass, and if CECT shows no enhancement of the mass, adrenal hemorrhage is more likely. The patient should be followed with USG for a change in size, and echo pattern. The mass representing the adrenal hemorrhage should decrease.[7,8] If the mass has a solid component, if color doppler USG reveals blood flow over mass, and if CECT shows enhancement of the mass, neuroblastoma needs to be ruled out. The patient should be carefully followed up with USG. In our patient, VMA levels were normal; there was no contrast enhancement on CECT abdomen, and follow-up USG revealed decrease in size of cyst.
